# Nudges and hard choices

**DOI:** 10.1111/bioe.13091

**Published:** 2022-09-24

**Authors:** Sarah Zoe Raskoff

**Affiliations:** ^1^ Department of Bioethics National Institutes of Health Bethesda Maryland USA

**Keywords:** autonomy, choice architecture, incomplete preferences, nudges

## Abstract

Nudges are small changes in the presentation of options that make a predictable impact on people's decisions. Proponents of nudges often claim that they are justified as paternalistic interventions that respect autonomy: they lead people to make better choices, while still letting them choose for themselves. However, existing work on nudges ignores the possibility of “hard choices”: cases where a person prefers one option in some respects, and another in other respects, but has no all‐things‐considered preference between the two. In this paper, I argue that many significant medical decisions are hard choices that provide patients with an opportunity to exercise a distinctive sort of “formative autonomy” by settling their preferences and committing themselves to weigh their values in a particular way. Since nudges risk infringing formative autonomy by depriving patients of this opportunity, their use in medical contexts should be sensitive to this risk.

## 1 NUDGES: THE BASIC CASE

One aim of medical care is to benefit patients: to restore the health of those who are ailing from sickness or disease and leave them better off than they would otherwise be. This idea is captured by the principle of beneficence.^1^ But there is a general consensus, in both medical ethics and the law, that the principle of beneficence is constrained by the principle of autonomy.^2^ Most people know what they value and what sorts of tradeoffs they are willing to make, say, between comfort and longevity; they have the capacity to assess the options available to them and to make decisions that align with their values. While healthcare providers should try to improve the health and welfare of their patients, they must do so in a way that respects a patient's capacity to decide for themselves based on their values: they must respect their patients' autonomy or capacity for rational choice.

But recent findings in behavioral economics call into question the extent to which people, left to their own devices, succeed in autonomously or rationally choosing for themselves.^3^ The trouble is that we are less capable of assessing and choosing options that align with our values or preferences than philosophical discussions of autonomy typically presuppose. For example, if you inform a patient that a medical operation has a 10% chance of failure, they are significantly less likely to agree to it than if you inform them that it has a 90% chance of success. This is puzzling because these two statements are logically equivalent and a perfectly rational person would treat them as such. But we are not perfectly rational. The presentation of options affects our evaluation of choices and the decisions we make. More generally, we are subject to many biases that predictably influence decision making: for example, we tend to assign greater importance to present rather than future gains and losses, to be more averse to losses than we are keen on gains, and to privilege the current state of affairs or status quo.^4^ Since different biases may be stronger or weaker depending on how options are presented to us, our choices significantly depend on the presentation of options—on what some call “choice architecture”—and often in surprisingly impactful ways.^5^

Recognizing the effect of choice architecture on decision‐making has led many to endorse the use of “nudges.”^6^ A nudge is a small change in the choice architecture that makes a predictable impact on the decisions we make. A classic example of a nudge is changing the location of food items in a cafeteria so that healthier items are at eye level. Healthy options are better for you, and many people sincerely claim to value eating healthier but, perhaps due to weakness of will, fail to make food choices that align with or promote this value when the time comes to eat lunch. It turns out, however, that simply making healthy options more salient significantly affects the choices people make: people are more likely to choose healthy food when it's right in front of them. So nudging people in this way seems to benefit them without compromising their autonomy—they are still deciding for themselves, and now in alignment with their own values or preferences. This strikes many as morally better than more restrictive ways of influencing choice, such as significantly raising the price of unhealthy foods or removing them from the cafeteria altogether, which intuitively infringe autonomy by restricting the menu of options. If someone really wants to eat something unhealthy, the option is still available.

Nudges, then, are supposed to be an effective and morally acceptable way of influencing choice. Some nudges benefit third parties, for example, nudging people into being organ donors by changing the default option: people retain the choice not to be organ donors, but have to “opt‐out” rather than “opt‐in” to organ donation when filling out forms for their driver's license. But most paradigmatic nudges, and the sorts of nudges I will focus on here, are intended to benefit the person who is nudged. Thaler and Sunstein call such nudges “libertarian paternalist.”^7^ These nudges are libertarian because they do not infringe autonomy, but paternalist because they benefit the person who is nudged. In the medical context, such nudges seem to provide a way to thread the needle between the principle of beneficence and the principle of autonomy. If physicians want to benefit their patients while still respecting their autonomy, nudges seem the perfect choice.

## 2 HARD CHOICES: THE PROBLEM

The case for certain nudges seems unassailable. Automatically scheduling a patient's medically necessary follow‐up appointments but leaving open the choice to decline is a good way to increase the odds that the patient returns for further medical care. This benefits them and does not infringe on their autonomy. If anything, such nudges may even promote autonomy, at least on the reasonable assumption that the patient prefers to receive the treatment in question but might absentmindedly forget to follow up. The exercise of autonomy, I will assume, involves choosing for oneself, and in line with one's own values or preferences. Nudges leave a person free to choose for themselves, and well‐implemented nudges can help align a person's choices with their values, leading them to make better choices.

But nudges are more worrisome in the absence of this alignment. Most obviously, a nudge might lead someone to make a choice that is contrary to or incompatible with their values. For example, it isn't justifiable—at least on libertarian paternalist grounds—to nudge someone into a treatment option that will prolong their life with extremely severe side effects, when they would prefer to die with dignity. Such a nudge makes it less likely that a person's choice reflects or promotes their values. And this seems problematic, at least on the assumption that respecting someone's autonomy means enabling them to make a choice that is the best option overall, given their values.

Another way a nudge might conflict with autonomy, however, has not been adequately appreciated. A nudge might lead someone to make a choice that isn't incompatible with their values, but rather is underdetermined by them in the sense that the options available relate to the person's values in an interesting way: the person prefers one in some respects, the other in other respects, but has no all‐things‐considered preference between the two. Following Levi and Chang, we might call decisions with this structure “hard choices.”^8^ What is special about hard choices isn't merely that the chooser prefers each option in certain respects, but rather that they have no settled tradeoff rate between those respects. They, therefore, have no all‐things‐considered preference between their options; this is what makes their choice hard.

At least in an important class of cases, when someone is confronted with a hard choice, nudging them one way rather than another looks different than it does in cases where one option either aligns with that person's values or else is incompatible with them.^9^ Here, the idea that respecting someone's autonomy requires enabling them to make a choice that better aligns with their values is unhelpful because, by hypothesis, the decision is such that our chooser's values do not specify one alternative as best. One aim of this paper is to suggest that medical contexts often involve hard choices of this sort and that these hard choices are often furthermore high stakes or *momentous* in the sense that they involve choices about values that are deeply personal and important. The second aim is to suggest that when it comes to momentous choices, choosing autonomously requires reflecting on the decision, “settling” the tradeoff between one's values, and so arriving at an all‐things‐considered preference. I call this kind of autonomy “formative autonomy” and argue that nudges sometimes interfere with it in an ethically objectionable way.

Here is the basic idea. When faced with momentous choices, patients do not have a settled (all‐things‐considered) preference that a well‐placed nudge can promote. What makes the choice hard, after all, is precisely that the person's values do not resolve the question of what option they most prefer. In such cases, to choose an option is to choose or settle one's preferences and so commit oneself to being someone who values in a particular way. Choosing not only options that better satisfy our preferences, but also our very preferences themselves is a distinctive exercise of autonomy. Nudging people in these contexts can infringe their autonomy by precluding them from autonomously settling their preferences, in a way that nudging them into taking the means to their ends does not. Since medical contexts are rife with momentous choices, we must be sensitive to them when designing choice architecture in medical settings.

The rest of the paper will flesh out this basic line of thought. I'll begin by introducing two different accounts of decision making—the Standard and the Nonstandard Model—and explain how they relate to libertarian paternalism. I'll argue that while libertarian paternalists tend to adopt the Standard Model, it cannot accommodate the possibility of hard choices. The Nonstandard Model can make sense of hard choices, and so makes space for an important exercise of autonomy: the act of settling one's preferences. Next, I'll explain why nudges threaten this sort of formative autonomy and make plausible the claim that medical contexts often involve choices that are not only hard but momentous. Nudges are an important tool and my suggestion isn't that we abandon them in medical contexts, but merely that the standard way of thinking about when nudges are justified ignores an important aspect of autonomy.

## 3 THE STANDARD MODEL

In the next two sections, I'll contrast two ways of thinking about autonomous choice, focusing exclusively on those who are sufficiently informed and meet the requisite clinical standards to count as capacitated.^10^ A key idea here is a “preference.” To say that someone “prefers” option A to option B is to say that they take A to be more desirable or choiceworthy than B, where I understand preferences as describing all‐things‐considered verdicts between options. Such all‐things‐considered preferences often depend on a person's plurality of more specific values and their tradeoff rates between them: someone may prefer A to B because A better promotes one value, B better promotes another, and one finds the former weightier in this context. Preference, then, is comparative: it describes a relation between options. On the Standard Model, for any domain of options, we can speak of a person's “preference ordering” over those options. Crucially, an ordering is *complete*: for any two options A and B, a person either prefers A to B, prefers B to A, or is indifferent between A and B in the sense that they deem A and B exactly equally good.

The Standard Model provides a straightforward picture of rational and autonomous choice. Starting with rationality, suppose Alex is out to brunch with friends and is mulling over available entrees. The restaurant has two options: pancakes and oatmeal. The two options cost the same, but Alex cares about both taste and health, and while the oatmeal is healthier, she finds pancakes tastier. So what should she choose? Well, according to the Standard Model, the answer is simple: whichever she prefers. Alex, by hypothesis, has a complete preference ordering over any options she encounters which is generated by her settled tradeoff rates between her values—in this case, the tradeoff between taste and health. So, for example, it may be that Alex cares enough about health that the difference in health between pancakes and oatmeal outweighs the difference in tastiness between the two, and she prefers the oatmeal. Then, she rationally ought to choose the oatmeal.

In this model, then, Alex chooses rationally when she chooses what she most prefers from the options available: choosing rationally just is choosing what one most prefers, given one's settled preferences. Now, not everyone is so rational: for example, Alex might choose the pancakes out of weakness of will, or because they are more salient. In these cases, a well‐designed nudge might help Alex to do the rational thing and order the option she already prefers. This does not infringe Alex's autonomy, because it does not infringe the exercise of her capacity for rational choice—in fact, it improves it, by helping her to choose the option she already most prefers, and so would choose if she didn't suffer from weakness of will or salience bias. Since healthy food is in fact better for Alex, this nudge counts as libertarian paternalist: it benefits her without infringing her autonomy or capacity for rational choice.

This isn't to say that *any* way of influencing people's choices to get them to choose more preferred options counts as libertarian in this sense. For example, suppose that instead of nudging Alex into getting the oatmeal, the restaurant owner influences her choice either by removing the option to order pancakes or by making the pancakes very expensive (thus removing the option of ordering cheap pancakes). Some see this as an infringement of autonomy, because, in many views, autonomous choice involves not only rationally choosing one's most preferred option, but also choosing from a sufficient range of options.^11^ In the case of ordering breakfast, this might seem trivial, but in other cases, the concern is more significant. For example, in a medical context, suppose a doctor is positive that a treatment would not only benefit their patient but also that the patient would in fact prefer to receive it. It still seems important that the doctor give the patient the opportunity to decide whether to receive it. Intuitively, it violates autonomy to force people to choose particular options, or to deprive them of the opportunity to choose alternative options, even if this channels them to more preferred options.

We can now see why libertarian paternalism seems so attractive. On the Standard Model, a choice is autonomous so long as it's both rational and (perhaps) made from a sufficient range of options. Violations of autonomy therefore either involve inducing people to choose dispreferred options or depriving them of options. Coercive forms of paternalism deprive people of options, but nudges are noncoercive, and so only violate autonomy if they cause people to choose dispreferred options. On this model, actively reflecting on one's options and deciding for oneself is therefore important only insofar as it leads one to choose options that one *already* prefers. Such reflection may be important because even if one comes to a decision with settled values and tradeoff rates between them, it isn't always obvious how to map these to one's options in order to determine one's preferences: one might lack self‐knowledge or empirical information relevant to evaluating one's options (for example, one might have a settled tradeoff rate between health and tastiness, but not appreciate how much one prefers the taste of pancakes or realize how much healthier oatmeal is). But importantly, so long as nudges don't lead one to choose dispreferred options, and don't deprive one of options, there is simply no room to worry that they might infringe one's autonomy.

## 4 THE NONSTANDARD MODEL

Although there are many ways one might depart from the Standard Model, for our purposes, only a slight departure is necessary. We may continue to assume that people have settled values and that they sometimes have settled tradeoff rates between them yielding (all‐things‐considered) preferences over options. However, the key difference is that we no longer assume that these preferences are complete: in some cases, a person prefers A to B in some respects, prefers B to A in others, but has no all‐things‐considered preference between the two (nor is the person all‐things‐considered indifferent between them). When this occurs, a person faces what we earlier called a “hard choice.”

It might seem like there are no genuine cases of hard choices and that their distinctive difficulty is merely apparent: either the alternatives are exactly equally good, in which case the chooser is simply indifferent, or else the chooser is missing information about the alternatives. To see why this isn't always so, consider a classic example of a hard choice: deciding between two career options. Should you be a doctor or a philosopher? Well, there are pros and cons on each side. For example, doctors get to interact with patients and their families and make a real, concrete impact on their lives. You know that you would find this rewarding and that you would also benefit from the higher salary. But philosophers do not have to make life‐or‐death decisions and have more freedom to explore whatever questions they find interesting and more flexibility in how they spend their time. This, too, appeals to you. So, which career should you choose?

Sometimes even significant life choices like this are relatively easy: after weighing the considerations, one finds that one indeed prefers one option to the other. But other times, even after careful deliberation and even after gathering all relevant information, neither option prevails. Although there may be some cases where this occurs because one is perfectly indifferent—the considerations on each side exactly balance out—more often this kind of case seems to involve incomplete preferences.^12^ The test for this is whether a very small improvement to one option would tip the balance and lead one to prefer that option.^13^ For example, would one come to prefer to be a doctor if the salary were raised by 1%? If one were perfectly indifferent beforehand, this slight increase in salary would decide the case: you would now prefer to be a doctor. But there certainly seem to be many cases where small improvements are not decisive in this way. In these cases, one faces a genuinely hard choice: one does not prefer A to B, or B to A, but neither is one indifferent between the two.

You might think that the rational response to a hard choice and to indifference is one and the same.^14^ If one is indifferent between A and B, one should just arbitrarily pick one—perhaps simply going for the most salient option or flipping a coin. But this doesn't seem like the right response to a hard choice. Return to our case of deciding on a career. Thinking that one should simply flip a coin to decide whether to apply to medical school or graduate school in philosophy does not do justice to the difficulty of the choice and all that hangs in the balance. Of course, if everything exactly balanced out, such that one were perfectly indifferent between the considerations on either side of the decision, then flipping a coin would be fine: each option would be exactly equally good. But this isn't how hard choices present themselves. If they could be rationally or autonomously treated the same way as cases of indifference, they would not present to us as *hard*. But they do.

How, then, is one to resolve hard choices? Following Chang, I propose that we resolve hard choices through a distinctive exercise of rational agency.^15^ When our accepted values and tradeoff rates between them do not settle our preference between A and B, the rational and autonomous thing to do is to settle one's preferences by deciding how to tradeoff the relevant values. One arrives at a hard choice without a settled tradeoff rate between, say, having a higher salary and having more flexibility. To choose autonomously in such a case, one must first settle which of these considerations one values more, and only then should one choose the option that better aligns with one's values. We might put this by saying that while the Standard Model of autonomous choice only makes room for *decisional* autonomy—that is, the sort of autonomy involved in choosing your most preferred options (from a suitable menu)—the Nonstandard Model makes room for what I call *formative* autonomy—that is, the sort of autonomy involved in settling the tradeoff rates between one's values and so settling one's preferences. And since our identity significantly depends on our values and the weights we assign them, formative autonomy is important: it involves choosing the sort of person that one is or will be.

The Nonstandard Model, then, opens the door to a different way we might violate someone's autonomy. Insofar as it seems important to our formative autonomy that we actively settle our preferences for ourselves when faced with hard choices, we might respect someone's decisional autonomy yet violate their formative autonomy. And, as I will now argue, even granting defenders of nudges that they leave decisional autonomy intact, their adherence to the Standard Model blinds them to the effect of nudges on formative autonomy. In the next section, I elaborate this point. In the following section, I suggest that this is an especially important consideration in medical contexts.

## 5 NUDGES AND FORMATIVE AUTONOMY

It might seem hyperbolic to say that we are at risk of objectionably violating an important aspect of someone's autonomy whenever they face a hard choice. Return to Alex's breakfast decision between pancakes and oatmeal, but suppose now this is a hard choice: Alex prefers pancakes in one respect, oatmeal in another, but has no all‐things‐considered preference between the two. Now suppose the restaurant owner decides to implement a two‐menu policy to nudge patrons into making healthier breakfast decisions. The default menu only lists healthy options, but diners who wish to order something more indulgent can request the supplementary menu. A two‐menu policy might very well undermine Alex's formative autonomy by leading her to order oatmeal without first actively settling the conflict between her values for health and taste, or by settling this conflict for her. But even granting as much, this does not seem cause for serious concern. When it comes to “mundane” hard choices of this sort—choices where the stakes of the decision are sufficiently low—it does not seem particularly objectionable to infringe formative autonomy by way of a nudge. Breakfast decisions are paradigmatically mundane choices.

But some hard choices have decidedly higher stakes, and when they do, violations of formative autonomy seem more troubling. Consider again a hard career choice: doctor or philosopher? In a case of this sort, where one's choice will make a significant impact on the sort of person one will be or the life one will lead, it's important that one settles one's values for oneself. Let's say—again, following Chang—that a person “drifts” into a career as a doctor if they pick that career, but without first settling on a preference for being a doctor over a philosopher.^16^ They choose to be a doctor, not because of a decision about what to value or what kind of person to be, but rather, say, because it feels like the path of least resistance. Such a person lives a less thoroughly autonomous life than someone who has committed to a career as a doctor, and “thrown their agency” behind the decision. This may come out in the sense of alienation they feel from themselves and their own life pursuits: they never really settled on being a doctor, and so were not able to rationally decide to become one, but rather simply found themselves with this identity and career. And this may remain true even if, after drifting into being a doctor, they develop a preference for being one, but only as a result of an adaptive preference for their new status quo.^17^ In this case, the cost to their autonomy comes not from the fact that they lack a settled preference, but rather from the fact that they never settled their preferences for themselves. Their preferences were settled by external forces, and they never had a chance to exercise their formative autonomy. They never decided how to weigh their values or what kind of person to be.

Let us say that a choice is “momentous” if it's both a hard choice and one that is high stakes in the sense described above (that is, it isn't “mundane”). Although the precise boundary between mundane and momentous choices is difficult to specify, as we have just seen, there are many clear cases of hard choices falling on the momentous side of the boundary. When it comes to such momentous choices, it's important to respect someone's formative autonomy by allowing them to settle their preferences for themselves, or even by facilitating the conditions under which they can do so. Nudges and other interventions designed without this in mind might seriously infringe formative autonomy.

We can bring this out by imagining that someone facing a momentous career choice is nudged one way or the other. Suppose someone deciding whether to be a doctor or a philosopher attends a counseling meeting with her academic advisor. At the meeting, she learns that her advisor has taken it upon himself to pre‐register her for pre‐med courses next year. He also provides pamphlets with information about how to study for the MCAT and which medical schools have alumni on their admissions committees. When she mentions that she loves philosophy and is also seriously considering a career as a professor, he says that it's her choice, and if she wants, she can cancel her enrollment in the pre‐med track and register for philosophy, giving up a career in medicine in pursuit of a career in academia. In other words, throughout the entire discussion, he presents a career in medicine as the default option she would be losing out on if she decided to opt‐out of it, and a career in philosophy as something that she might elect to opt‐in to if she wishes to pursue a different path.

The academic advisor employs two well‐known sorts of nudges: one involving people's tendencies to stick with the default option, and another involving the tendency to be averse to options that are presented as losses. Consider, for example, that he might have instead pre‐registered her for philosophy classes yet told her it was up to her whether she wishes to opt‐out and so lose her chance to pursue a career in philosophy. Such nudges are surprisingly effective, and might very well cause the individual in question to stick with the path of least resistance and drift into the career she is nudged into, without settling for herself what she really prefers. This seems like a problematic infringement of her formative autonomy. A different, better academic advisor would listen to his advisee, and encourage her to take the time to carefully weigh her options and settle her preferences for herself.

We can imagine two problematic outcomes in this case. First, the advisee might never settle their preferences, and so find themselves in a career they do not prefer, never having settled whether their other career path would better align with their values. Second, they might eventually come to prefer their life as a doctor through a process of preference adaptation. In this latter case, the cost to formative autonomy is arguably greater. Rather than allowing the student to settle her own preferences, the academic advisor managed to impose preferences on her and settle them on her behalf. One can hardly think of a more paradigmatic violation of autonomy than others imposing their ends or preferences on you—a clear case of “heteronomy.”^18^

To be clear, I am not arguing that it's always bad for someone that they drift—or even for their preferences to be settled for them by some external agent. Causing someone to drift is morally objectionable, when it is, not because it necessarily makes the person *worse off* but because of its effects on the drifter's autonomy. Settling one's preferences is an exercise of formative autonomy. When one causes someone to drift, one deprives them of an opportunity to exercise their autonomy (or at least steers them away from this opportunity), and so causes them to lead a less thoroughly autonomous life. Even if this isn't as severe an infringement as the case where one imposes a new preference on them, it's still an infringement. Of course, this isn't to say that autonomy, or specifically formative autonomy, must take priority in bioethical analysis: sometimes infringing autonomy may be, all‐things‐considered, what we ought to do. My claim is that nudges are typically justified on the ground that they influence choice without infringing autonomy, and for an important class of cases—momentous choices—this isn't true.

Before going on, it's worth noting how the recognition of formative autonomy not only makes room for this new way that nudges might violate autonomy but also undercuts a common defense of nudges. Some people defend nudges on the grounds that they are only likely to work in cases where people lack strong preferences to begin with, such that they are unlikely ever to lead individuals to choose strongly dispreferred options.^19^ For example, if our advisee strongly preferred a career in philosophy, then her advisor's nudges would not be successful—she would be more resistant to them. This “resistibility” defense of nudges makes sense on the Standard Model on which people have complete preferences such that nudges can violate autonomy only by causing them to choose a less preferred option or by restricting the options from which they choose. But it no longer makes sense once we realize that cases in which people lack strong preferences might involve hard choices rather than (near) indifference. Indeed, we might worry that precisely because people lack preferences in contexts of hard choices, they are especially susceptible to nudges. But this clearly isn't an adequate defense of nudges, once we realize that nudging people in cases where they lack preferences may violate their autonomy in a way that nudging them in cases of indifference does not. Rather, if it's true that people are more resistant to nudges when they have a strong preference against the option they are being nudged into, this suggests that nudges may be especially likely to violate formative autonomy in the context of hard (and specifically momentous) choices, where they lack preferences altogether. The resistibility defense gets things backwards in contexts of momentous choice.

## 6 MOMENTOUS CHOICES IN MEDICAL CONTEXTS

I have argued that nudges risk violating formative autonomy in contexts of momentous choice. What remains to be seen is why this is especially worrisome in medical settings. My aim in this section is to elucidate this worry by considering several choices patients face in medical settings that qualify as momentous in the sense characterized above. These are cases where patients must make high‐stakes choices about their clinical care, but where it seems especially likely that they have incomplete preferences over their options: they have conflicting values that may not determine one option as best. In such cases, deciding requires exercising one's formative autonomy: resolving preference incompleteness by settling the weights of one's values, settling one's preferences, and so deciding what kind of person to be. So, in designing nudges for use in medical contexts, we must be especially sensitive to the existence of momentous choices. Unfortunately, existing work on nudges in medical settings seems to overlook this worry.

Imagine a woman, call her Kat, who books an appointment with her gynecologist after experiencing some unusual discomfort and cramping. After a series of tests, the doctor reveals to Kat that she is pregnant. Kat is ambivalent. She loves children but now doesn't seem to be the right time, while she is still completing her degree. She has a loving partner, but they never imagined having children—if ever—before getting married and settling down into stable jobs. Still, when she thinks about having a baby, she feels happy and tender and excited. And even though Kat fiercely believes that people should have the right to decide whether to terminate a pregnancy, deep down she feels some moral compunction about having an abortion herself.

Kat must decide whether she wants to continue her pregnancy, and there are clearly many relevant considerations pulling her in both directions. Kat values her education and the prospect of a fulfilling and rewarding career. But she is also drawn to the idea of becoming a mother and has some moral reservations about having an abortion. In weighing up these various considerations, it seems reasonable to assume that at least sometimes, people in Kat's circumstances find themselves genuinely unable to regard one option as all‐things‐considered better than the other. And it seems furthermore reasonable that many such cases do not involve indifference: it isn't as if the decision would become straightforward and easy if we made one option slightly more attractive, for example, by slightly decreasing the monetary cost of abortion or childcare. People in Kat's position therefore sometimes face hard choices in the sense that they have incomplete preferences over their options. And these choices are clearly momentous: they are high stakes, making a significant difference to the life that person will lead, or the kind of person they will be. Kat might therefore face a momentous choice that provides an opportunity for her to exercise her formative autonomy. Before she can decide whether to continue her pregnancy, she must settle her preferences by determining what she values most.

This is just one example of a medical context where momentous choices are likely sometimes to arise. The basic algorithm for identifying such contexts is to look for cases where (1) there are important, high‐stakes considerations on both sides of a decision and (2) when patients have difficulty deciding between their options, the difficulty isn't always resolved by slightly improving one of their options—for example, by making the option slightly less expensive. This is because momentous decisions are hard choices with high stakes, and condition (1) ensures that the choice is high stakes while condition (2) ensures that it's sometimes hard. And sure enough, there are many medical contexts satisfying both conditions. Consider the following partial list: patients must decide whether to continue with invasive chemotherapy that is unlikely to work and live in pain and discomfort or to die comfortably in hospice care; whether to undergo risky but ultimately cosmetic surgery; whether to donate a kidney; whether or how to medically transition in light of gender dysphoria; whether to accept a life‐saving treatment that violates strongly held religious convictions; whether to get a cochlear implant later in life; whether to participate in experimental research with no prospect of direct benefit, and so on. Each of these decisions involves important considerations on each side. Each of these examples involves a decision that is often difficult in the colloquial sense, and where this difficulty isn't always resolved by slightly improving one of the options, suggesting that such choices are indeed sometimes hard in the technical sense defined above. Medical contexts are rife with momentous choices.

The connection to nudges is obvious. In many of the medical contexts in which I have just suggested momentous choices are likely to arise, we have already begun to nudge patients without thinking about any of this. Consider, for example, nudges designed to steer reproductive choices. In the wake of *Planned Parenthood v Casey*, several regulations that influence the right to choose an abortion have been proposed and upheld on the basis that they do not prohibit a person from exercising this right, but rather ensure that their consent is appropriately informed and the product of careful deliberation. Examples of such regulations include requirements that people seeking abortions observe a waiting period, receive information about fetuses' moral or legal status, view an ultrasound, and listen to a fetus' heartbeat.

This case is complicated since the stated justification for many of these nudges is that they give people time to reflect seriously on their decision and ensure that the decision is voluntary. However, in practice, many of these nudges seem instead to set the default as continuing a pregnancy. To my knowledge, there are no states requiring someone to deliberate carefully for 24 h or to read about or watch videos depicting how hectic their life will become once they have children before deciding *not* to terminate their pregnancy, nor do states mandate providing information about the risks of continuing a pregnancy and giving birth (which are generally significantly higher than early abortion).^20^ My point here, again, isn't to argue that all nudges are ethically problematic, but rather that we need to be careful thinking about the effect of nudges on formative autonomy in contexts of momentous choice. And it seems safe to say that whether to become a parent is often a momentous choice.^21^

Another nudge generating this concern pertains to patients' choices about end‐of‐life care. Halpern and colleagues have done fascinating work on the effect of default options on advance care planning documents, testing whether even deeply personal and important choices can be influenced by defaults.^22^ And it turns out that they can: defaults strongly affect the treatment choices seriously ill patients make when completing advance directives. Patients completing advance directives in which comfort‐oriented care was the default were significantly more likely to choose comfort‐oriented treatment options than were patients completing advance directives without specified defaults. And patients completing advance directives in which life‐extending care was the default were significantly more likely to choose life‐extending care.^23^

Halpern et al. suggest that these results “raise the possibility that people might not have strongly held views on what forms of care at the end of life best promote their values.”^24^ In a follow‐up paper, Gorin et al. argue that this is further confirmed by the fact that even after being debriefed about the manipulation of the default, “no patients revised their choices”: “defaults do not push people in directions they would strongly prefer not to go” because “if they did, people would either override the default initially or more frequently revise their nudged choices.”^25^ Since patients don't do this, they conclude that “the emerging evidence [is] that defaults do not necessarily override patient preferences and that, on reflection, patients tend to endorse their nudged choices.”^26^ The normative implication they draw is that, in cases where patients appear to lack settled preferences in this way, nudges are especially appropriate: “when patients lack authentic preferences over their treatment options and are therefore likely to make important decisions under the influence of trivial and irrelevant features of their choice environment, clinicians and policy‐makers are justified in using nudges in accordance with the best‐interest standard.”^27^

Gorin et al.'s treatment of nudges is unusually thoughtful, insofar as it acknowledges that patients may have incomplete preferences over their options. I single them out only because their analysis of cases of incomplete preferences clearly exemplifies the failure of choice architects to acknowledge the existence and importance of formative autonomy. Gorin et al. believe that, in cases of incomplete preferences, there can be no serious objection to nudges, since they don't conflict with patients' preferences. But the argument of this paper has been that incomplete preferences provide opportunities to exercise formative autonomy, and so should make us wary of, rather than enthusiastic about, implementing nudges. It's precisely because patients lack settled preferences about what end‐of‐life care option best promotes their values that nudging them risks objectionably violating their autonomy. As we have already seen, claiming that “defaults do not push people in directions they would strongly prefer not to go” isn't a good defense of nudging in circumstances of momentous choice, where it seems especially important that people settle their preferences for themselves.

That said, Gorin et al. also emphasize the importance of supplementing nudges with processes of joint decision‐making between physicians and patients. This aspect of their discussion is highly welcome. It's true that physicians can help patients make momentous choices by providing them an opportunity for reflecting as well as the information they need to weigh their values and settle their preferences. And insofar as we can nudge patients into making momentous choices for themselves, this may be a way of enhancing formative autonomy: for example, a patient who might otherwise be likely to drift down the path of least resistance might be nudged into stopping and reflecting on the values underlying their decision and how they wish to settle the corresponding tradeoffs. Indeed, as Manz et al. have shown, it's sometimes possible to nudge *physicians* into having “difficult conversations” with patients, of the sort that can empower patients to exercise their formative autonomy in making momentous choices.^28^

So, again, the upshot isn't that we should abandon nudges. First, nudges may be perfectly appropriate in a wide class of decision contexts where we have no reason to think that patients face hard choices, let alone momentous ones. For example, nudging patients into eating healthy food is not plausibly a significant violation of their formative autonomy (since even if the choice is hard, it's not momentous), nor is nudging them into getting cheaper but equally effective generic drugs rather than the brand name alternative (since this isn't plausibly a hard choice). Second, neither autonomy in general, nor formative autonomy specifically, is the only moral consideration relevant to evaluating the use of nudges. So even in cases when nudges violate formative autonomy, they may be all‐things‐considered justified, say, because of the extent to which they promote a patient's interests or broader social benefits. I've taken no stand on how to weigh such competing considerations here, but have merely argued that we make a serious moral mistake if we follow the standard model and so ignore the potential for nudges to violate formative autonomy.

Finally, in contexts of momentous choice, it may be possible to nudge patients in ways that *promote* formative autonomy by encouraging patients to make such decisions for themselves—although further work on this sort of nudge is needed. For example, although above I expressed some reservations about the use of mandatory waiting periods and ultrasounds to shape reproductive decisions, my misgivings about these nudges are based primarily in their asymmetrical use to steer pregnant people away from obtaining an abortion. In other contexts, nudging patients to reflect seriously on momentous choices, or nudging healthcare providers to facilitate such reflection, may be an effective way of promoting formative autonomy by encouraging patients to contemplate their options before deciding.^29^ And this, by the way, provides a response to what is sometimes called the “inevitability” defense of nudges, namely, that choice architecture is unavoidable so there is no point worrying about it. Some choice architecture is indeed unavoidable, but it can nevertheless be designed or shaped in better or worse ways, for example, in ways that either promote or violate, formative autonomy. My worry only arises, then, in cases where we are trying to nudge patients who face momentous choices in a particular way: into choosing without first settling their preferences for themselves.

## 7 CONCLUSION

Nudges have made their way into medical settings, and it's easy to see why. Healthcare providers should conform to the principle of beneficence: they should promote their patients' health and welfare. At the same time, they should respect patient autonomy, and allow patients to make choices that align with their values for themselves. A thoughtfully implemented nudge can thread the needle between beneficence and autonomy by helping doctors steer their patients toward decisions that align with their own values without preventing them from choosing otherwise.

In many cases, enthusiasm for clinical nudges is warranted. But some nudges are more concerning—specifically, those involving momentous choices. In this paper, I've argued that medical contexts often involve such choices: they are high‐stakes choices requiring patients to choose between options over which they have incomplete preferences. In contexts of momentous choice, choosing autonomously requires settling our preferences and deciding which of our values to prioritize. Despite their other virtues, nudges can interfere with this process. This consideration has been overlooked in existing discussions of nudges in general, and clinical nudges specifically. Respect for autonomy requires that we not overlook it any longer.

## CONFLICT OF INTEREST

The author declares no conflict of interest.

## Footnotes

1 Beauchamp, T. L., & Childress, J. F. (2013). *Principles of biomedical ethics* (7th ed.). Oxford University Press.

2 Gillon, R. (2003). Ethics needs principles—four can encompass the rest—and respect for autonomy should be “first among equals.” *Journal of Medical Ethics*, *29*, 307–312.

3 Kahneman, D. (2013). *Thinking, fast and slow*. Farrar, Straus and Giroux.

4 Ibid.; Blumenthal‐Barby, J. S. (2016). Biases and heuristics in decision making and their impact on autonomy. *American Journal of Bioethics*, *16*(5), 5–15.

5 Thaler, R. H., & Sunstein, C. R. (2008). *Nudge: Improving decisions about health, wealth, and happiness*. Yale University Press; Kahneman, op. cit. note 3.

6 See especially Thaler & Sunstein, op. cit. note 5, but also Saghai, Y. (2013). Salvaging the concept of nudge. *Journal of Medical Ethics*, *39*(8), 487–493.

7 See Thaler & Sunstein, op. cit. note 5; Thaler, R. H., & Sunstein, C. R. (2003). Libertarian paternalism. *American Economic Review*, *93*(2), 175–179.

8 Levi, I. (1986). *Hard choices: Decision making under unresolved conflict*. Cambridge University Press; Chang, R. (2002). The possibility of parity. *Ethics*, *112*(4), 659–688; Chang, R. (2012). Are hard choices cases of incomparability? *Philosophical Issues*, *22*(1), 106–126; Chang, R. (2017). Hard choices. *Journal of the American Philosophical Association*, *3*(1), 1–21.

9 On one definition, “nudge” is a success term: an intervention only counts as a nudge if it in fact leads someone to choose an option that they all‐things‐considered already prefer. Since hard choices involve the absence of such a preference, it follows, by this definition, that it is impossible to nudge those facing hard choices. However, both in practice and in the bioethical and philosophical literature, interventions (e.g., changes to default options) are widely described as nudges even if they do not fit this definition. Furthermore, such interventions are often defended as nudges and—as I will later argue—they have already been incorporated into a variety of circumstances that qualify as hard choices. I will therefore continue to employ the looser definition of a nudge as a small change in the choice architecture that makes a predictable impact on the decisions we make, even when such interventions do not successfully lead people to choose options they already all‐things‐considered prefer. Thanks to an anonymous referee for urging this clarification.

10 See Beauchamp & Childress, op. cit. note 1.

11 Raz, J. (1988). *The morality of freedom* (p. 172). Clarendon.

12 Ibid.; Chang (2002, 2012, 2017), op. cit. note 8.

13 Chang (2017), op. cit. note 8.

14 Sen seems to think so. Sen, A. (1997). Maximization and the act of choice. *Econometrica*, *65*(4), 745–779.

15 Chang (2017), op. cit. note 8; compare Blumenthal‐Barby, J. S. (2021) Ambivalence‐autonomy compatibilism. In B. Brogaard & D. Electra Gatzia (Eds.), *The philosophy and psychology of ambivalence: Being of two minds* (pp. 49–66). Routledge.

16 Chang (2017), op. cit. note 8.

17 For the classic discussion of adaptive preferences, see Elster, J. (1987). *Sour grapes: Studies in the subversion of rationality* (1st paperback ed.). Cambridge University Press, chapter 3.

18 Kant, I. (2002). *Groundwork for the metaphysics of morals*. Yale University Press.

19 For example, see the below discussion of Halpern, S. D., Loewenstein, G., Volpp, K. G., Cooney, E., Vranas, K., Quill, C. M., McKenzie, M. S., Harhay, M. O., Gabler, N. B., Silva, T., Arnold, R., Angus, D. C., & Bryce, C. (2013). Default options in advance directives influence how patients set goals for end‐of‐life care. *Health Affairs*, *32*(2), 408–417; Gorin, M., Joffe, S., Dickert, N., & Halpern, S. (2017). Justifying clinical nudges. *Hastings Center Report*, *47*(2), 32–38.

20 Raymond, E. G., & Grimes, D. A. (2012). The comparative safety of legal induced abortion and childbirth in the United States. *Obstetrics and Gynecology*, *119*(2 Pt 1), 215–219.

21 For a different analysis of a similar phenomenon, see Paul, L. A. (2014). *Transformative experience*. Oxford University Press.

22 Halpern, S. D., et al., op. cit. note 19.

23 Ibid.

24 Ibid: 2–3.

25 Gorin, M., et al., op. cit. note 19, p. 33.

26 Ibid. Here, Gorin, Joffe, Dickert, & Halpern distinguish between “authentic” and “constructed” preferences, describing the latter as “those that are (merely) produced through the process of elicitation.”

27 Gorin, M., et al., op. cit. note 19, p. 35.

28 Manz, C. R., Parikh, R. B., Small, D. S., Evans, C. N., Chivers, C., Regli, S. H., Hanson, C. W., Bekelman, J. E., Rareshide, C. A. L., O'Connor, N., Schuchter, L. M., Shulman, L. N., & Patel, M. S. (2020). Effect of integrating machine learning mortality estimates with behavioral nudges to clinicians on serious illness conversations among patients with cancer: A stepped‐wedge cluster randomized clinical trial. *JAMA Oncology*, *6*(12), 1–7.

29 Ibid. Thanks to an anonymous referee for encouraging this elaboration.

